# Collagen reorganization at the tumor-stromal interface facilitates local invasion

**DOI:** 10.1186/1741-7015-4-38

**Published:** 2006-12-26

**Authors:** Paolo P Provenzano, Kevin W Eliceiri, Jay M Campbell, David R Inman, John G White, Patricia J Keely

**Affiliations:** 1 Department of Pharmacology, University of Wisconsin, Madison, WI, USA.; 2 Molecular Biology Program, Laboratory for Optical and Computational Instrumentation, University of Wisconsin, Madison, WI, USA.

## Abstract

**Background:**

Stromal-epithelial interactions are of particular significance in breast tissue as misregulation of these interactions can promote tumorigenesis and invasion. Moreover, collagen-dense breast tissue increases the risk of breast carcinoma, although the relationship between collagen density and tumorigenesis is not well understood. As little is known about epithelial-stromal interactions in vivo, it is necessary to visualize the stroma surrounding normal epithelium and mammary tumors in intact tissues to better understand how matrix organization, density, and composition affect tumor formation and progression.

**Methods:**

Epithelial-stromal interactions in normal mammary glands, mammary tumors, and tumor explants in three-dimensional culture were studied with histology, electron microscopy, and nonlinear optical imaging methodologies. Imaging of the tumor-stromal interface in live tumor tissue ex vivo was performed with multiphoton laser-scanning microscopy (MPLSM) to generate multiphoton excitation (MPE) of endogenous fluorophores and second harmonic generation (SHG) to image stromal collagen.

**Results:**

We used both laser-scanning multiphoton and second harmonic generation microscopy to determine the organization of specific collagen structures around ducts and tumors in intact, unfixed and unsectioned mammary glands. Local alterations in collagen density were clearly seen, allowing us to obtain three-dimensional information regarding the organization of the mammary stroma, such as radiating collagen fibers that could not have been obtained using classical histological techniques. Moreover, we observed and defined three tumor-associated collagen signatures (TACS) that provide novel markers to locate and characterize tumors. In particular, local cell invasion was found predominantly to be oriented along certain aligned collagen fibers, suggesting that radial alignment of collagen fibers relative to tumors facilitates invasion. Consistent with this observation, primary tumor explants cultured in a randomly organized collagen matrix realigned the collagen fibers, allowing individual tumor cells to migrate out along radially aligned fibers.

**Conclusion:**

The presentation of these tumor-associated collagen signatures allowed us to identify pre-palpable tumors and see cells at the tumor-stromal boundary invading into the stroma along radially aligned collagen fibers. As such, TACS should provide indications that a tumor is, or could become, invasive, and may serve as part of a strategy to help identify and characterize breast tumors in animal and human tissues.

## Background

Tissue microenvironments play an important role in maintaining normal cell behavior [1-3]. Moreover, type I collagen is a prevalent component of the stromal extracellular matrix; its expression being spatially and temporally regulated during mammary ductal formation, suggesting it plays important roles in development [4]. Consistent with this idea, decreasing the levels of α_2_β_1 _integrin, a primary type I collagen receptor, disrupts mammary epithelial tubulogenesis in vitro [2] and alters branching morphogenesis in vivo [5], respectively. Furthermore, inappropriate stromal-epithelial interactions can promote tumorigenesis [6-8], and in breast cancer, metastatic epithelial cells migrate in direct contact along stromal collagen fibers [9]. The importance of studying stromal interactions in breast tissue is further reinforced by the fact that patients with collagen-dense breast tissue possess a greater than fourfold increased risk of breast carcinoma [10,11]. Although the mechanisms mediating the effects of the extracellular matrix (ECM) on breast carcinoma development in vivo are largely unknown, contributing factors may be adhesion mediated signaling and mechanical signals imparted on mammary epithelial cells from surrounding type-I collagen-rich stroma, either directly or across basement membrane proteins. One important step to elucidating these signaling interactions is to determine the organization of the collagenous stroma surrounding both normal mammary glands and tumors within intact tissue so as to better understand the cell-matrix interaction and how matrix organization, density, and composition affect tumor formation and progression.

Nonlinear microscopy techniques such as multiphoton laser-scanning microscopy (MPLSM) and second harmonic generation (SHG) provide powerful tools to image cellular autofluorescence and extracellular matrix structure in intact tissues [12-15]. Both techniques are well suited for high-resolution in vivo imaging, and second harmonic generation is particularly adept at imaging collagen structure. Specifically, multiphoton microscopy results from the nonlinear excitation of molecular fluorescence and can produce images deep into thick tissues [16,17], while SHG signals depend on non-linear interactions of illumination with a non-centrosymmetric environment (e.g. fibrillar collagen) that can provide submicron resolution [13-15]. The most commonly utilized multiphoton process is two-photon excitation (2PE) of fluorescence, in which two low-energy (usually near-infrared) photons simultaneously excite a fluorophore, which later decays to produce a single fluorescent photon of lower energy than the corresponding one-photon (half wavelength of 2PE) excitation [13,18,19]. In this 2PE process the fluorescence is dependent upon the square of the intensity (see Methods), producing optical sectioning that makes it equivalent to confocal imaging in terms of restricting excitation to the plane of focus, but facilitates a much greater effective imaging depth and better cell viability [16,20]. SHG imaging, on the other hand, does not arise from an absorptive process, but instead the laser field suffers a nonlinear, second-order, polarization when passing through certain ordered structures resulting in a coherent signal at exactly half the wavelength of the excitation [21]. Great utility arises from the fact that MPLSM and SHG can be implemented simultaneously in live tissue to provide complementary information and a powerful experimental and diagnostic tool.

The purpose of this study was to characterize collagen morphology in intact tissues so as to understand the structure-function relationship of epithelial- and tumor-stromal interactions in the mammary gland with particular emphasis on local tumor cell invasion during carcinoma progression. We used both MPLSM and SHG imaging, in conjunction with additional correlative microscopy techniques, to detect differences in local collagen density near normal glands and mammary tumors, and identify distinct collagen fiber organization around tumors, with characteristic collagen structures such as radially aligned collagen fibers associated with tumor-cell invasion. Identification and characterization of these collagen signatures sheds insight into the process of tumor cell invasion, and may serve a diagnostic capacity for determining the invasive potential of tumors.

## Methods

### Mouse mammary tissues and tumors

This study was approved by the institutional animal use and care committee and meets N.I.H. guidelines for animal welfare. To study non-tumor bearing mammary glands, tissue was obtained from B6129SF2/J mice or Col1a1^tmJae ^mice (The Jackson Laboratory, Bar Harbor, ME, USA). To study tumor-stromal interactions in intact tissue, two mouse breast tumor models were utilized: MMTV-Wnt-1 (colony founder mice provided by Dr. Caroline Alexander, University of Wisconsin, Madison, WI, USA) and MMTV-polyoma middle-T (abbreviated PyVT following the Jackson Laboratory title but is also commonly abbreviated as PyMT or PyV-MT; colony founder mice originally obtained from Jackson Laboratory were provided by Dr. Amy Moser, University of Wisconsin, Madison, WI, USA).

### Tumor explants and collagen gel culture

To study tumor-mediated collagen reorganization and tumor cell invasion in vitro, tumor explants were obtained and cultured in a manner similar to a previous report by Friedl and co-workers [22]. Small pieces of tumor were harvested from the central region of palpable PyVT tumors (that were confirmed by histology) with a 3 mm biopsy punch and were cultured in type I collagen gels. Following removal, tumors were rinsed in DMEM containing penicillin/streptomycin/fungizone solution (Cellgro, Herndon, VA). A single tumor explant was then cultured within a 2.0 mg/ml collagen gel (8.0 mg/ml rat-tail collagen solution (BD Biosciences, San Jose, CA) neutralized with 100 mM HEPES in 2 × PBS). Following Gel polymerization for 1 hr, the tumor explant containing collagen gels were released from the culture dish and floated in DMEM containing penicillin/streptomycin solution supplemented with 10% heat inactivated FBS. Imaging was performed on live (non-fixed) cells in intact three-dimensional collagen gels.

### Multiphoton microscopy and second harmonic generation

Multiphoton excitation (MPE) with MPLSM allows imaging of endogenous fluorophores from deep inside live biological tissues with the fluorescence excitation primarily restricted to the plane of focus due to a quadratic dependence on the laser light intensity and a low probability of multiple low-energy photons being absorbed outside the focal plane [16,17,23]. For the case of a pulsed laser excitation, the time averaged fluorescent intensity is a function of the molecular cross-section *δ*_2_(*λ*) and the square of the laser intensity, *I*(*t*)^2^, and can be expressed as [23,24]:

〈If,p(t)〉=δ2Pave2τpfp[π(NA)2hcλ]2     (1)
 MathType@MTEF@5@5@+=feaafiart1ev1aaatCvAUfKttLearuWrP9MDH5MBPbIqV92AaeXatLxBI9gBaebbnrfifHhDYfgasaacH8akY=wiFfYdH8Gipec8Eeeu0xXdbba9frFj0=OqFfea0dXdd9vqai=hGuQ8kuc9pgc9s8qqaq=dirpe0xb9q8qiLsFr0=vr0=vr0dc8meaabaqaciaacaGaaeqabaqabeGadaaakeaadaaadeqaaiabbMeajnaaBaaaleaacqqGMbGzcqGGSaalcqqGWbaCaeqaaOGaeiikaGIaeeiDaqNaeiykaKcacaGLPmIaayPkJaGaeyypa0dcciGae8hTdq2aaSbaaSqaaiabikdaYaqabaGcdaWcaaqaaiabbcfaqnaaDaaaleaacqqGHbqycqqG2bGDcqqGLbqzaeaacqaIYaGmaaaakeaacqWFepaDdaWgaaWcbaGaeeiCaahabeaakiabbAgaMnaaBaaaleaacqqGWbaCaeqaaaaakmaadmaabaGae8hWda3aaSaaaeaacqGGOaakcqqGobGtcqqGbbqqcqGGPaqkdaahaaWcbeqaaiabikdaYaaaaOqaaiabbIgaOjabbogaJjab=T7aSbaaaiaawUfacaGLDbaadaahaaWcbeqaaiabikdaYaaakiaaxMaacaWLjaWaaeWaaeaacqaIXaqmaiaawIcacaGLPaaaaaa@5915@

In Equation 1, *δ*_2 _is defined as a molecular cross section that represents the dependence for the probability of two-photon excitation on the square of photon density, *P *is the laser power, *τ*_*p *_is the laser pulse width, *f*_*p *_is the laser repetition rate, *NA *is the numerical aperture, *h *is Planck's constant, *c *is the speed of light, and λ is the wavelength.

In contrast to multiphoton excitation, which obeys the fundamental physical relationship of energy loss following excitation, SHG is a conserved polarization process that follows the relationship [21,25,26]:

P = *χ*^(1) ^* E + *χ*^(2) ^* E * E + *χ*^(3) ^* E * E * E + …     (2)

where the polarization (P) and electric field (E) are vectors, and the nonlinear susceptibilities, χ^(i)^, are tensors. Therefore, SHG arises from the laser field suffering a conserved nonlinear, second-order, polarization when passing through non-centrosymmetric ordered structures that is described by term 2 of Equation 2.

For MPE and SHG imaging of live unfixed, intact (not sectioned), non-stained glands, and tumor explants within collagen gels, as well as hematoxylin and eosin stained histology slides, we used an optical workstation [27] that was constructed around a Nikon Eclipse TE300. For live tissue imaging, twenty mammary tissues including nine from the Col1a1^tmJae ^strain (three each of wild type, heterozygous and homozygous), and tumors from Wnt-1 (n = 10, plus wild type controls) and PyVT (n = 20) mice were harvested and live tissue maintained in buffered media at 37°C. All tissues were imaged immediately following tissue harvest and a Ti:sapphire laser (Spectra-Physics-Millennium/Tsunami, Mountain View CA) excitation source producing around 100 fs pulse widths and tuned to 890–900 nm was utilized to generate both multiphoton excitation (cellular autofluorescence from FAD) and SHG. The beam was focused onto the sample with either a Nikon 40× Plan Fluor oil-immersion lens (N.A. = 1.4) or a Nikon 60× Plan Apo water-immersion lens (N.A. = 1.2). All SHG imaging was detected from the back-scattered SHG signal [26], and the presence of collagen confirmed in our tissues using fluorescence lifetime imaging microscopy, or FLIM [12], on the same optical workstation, as the SHG from collagen has no lifetime. Additionally, due to the fundamental differences between MPE and SHG signals, filtering can separate the emission signals. Using a 464 nm (cut-on) long pass filter, MPE was discriminated from the total emission while a 445 nm narrow band pass filters was used to separate SHG (filters from TFI Technologies, Greenfield, MA). For 3D imaging in intact tissues, 2D (x-y) images were acquired at various serial depths (z) into the samples.

Acquisition was performed with WiscScan [28] a software acquisition package developed at LOCI (Laboratory for Optical and Computational Instrumentation, University of Wisconsin, Madison, WI, USA). Image analysis for combined MPE-SHG was performed with ImageJ [29] and VisBio [30] software. Using ImageJ, differences in collagen density were quantified by measuring the area of collagen signal following density slicing from a constant threshold, and local and mean intensity were measured within these normalized areas. For TACS-1 image analysis additional surface rendering plug-ins for ImageJ were utilized. For TACS-2 and -3, ImageJ was used to quantify the collagen fiber angle relative to the tumor. The tumor boundary was defined and the angle relative to the tangent of tumor boundary was measured every 10 microns.

### Histology and electron microscopy

For histology, formalin-fixed paraffin-embedded samples from eight B6129SF2/J mice and eight Col1a1^tmJae ^mice were sectioned and stained for hematoxylin and eosin, trichrome, and picrosirius red using standard techniques. Additionally, all tissues imaged with MPLSM were subsequently fixed and processed for histology to confirm the presence of tumors and characterize the tumor morphology. Sample preparation for scanning and transmission electron microscopy (SEM and TEM, respectively) was performed by fixing whole mammary glands in 2.5% formaldehyde/2.5% glutaraldehyde in 0.1 M sodium cacodylate buffer for 1 hr at room temperature (RT), after which sample were placed in fresh fixative overnight at 4°C. Samples were then washed in cacodylate buffer and postfixed in 1.5% osmium tetroxide at RT for 1.5 hrs. Samples (SEM n = 8 glands and TEM n = 6 glands) from B6129 mice were again washed in buffered solution and dehydrated, fractured, critical point dried, sputter coated, and imaged with SEM, or stained, dehydrated and cleared, embedded, and then sectioned for imaging with TEM [31].

## Results

### The normal mammary gland

**Figure 1:**
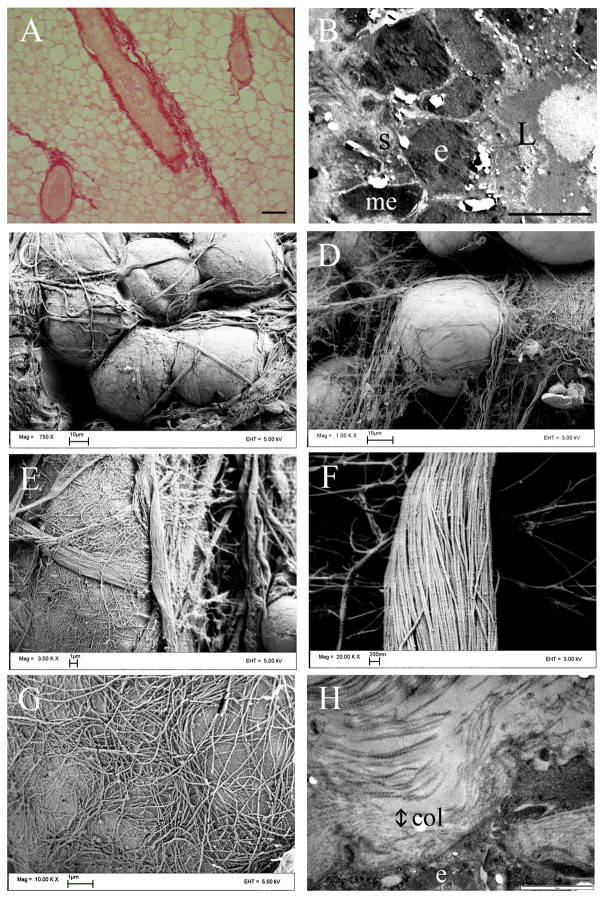
**Collagen morphology and the epithelial-stromal interaction in the mammary gland**. (A) Picrosirius red staining (red), a selective collagen stain, indicating the primary stromal component is collagen (bar = 50 μm). (B) Low magnification (bar = 10 μm) TEM image of a mouse mammary duct (transverse section) showing the organization of epithelial (e) and myoepithelial (me) cells outside the lumen (L) in close association with the collagenous stroma (s), with myoepithelial cells not completely separating the stroma from the epithelial cells. (C) SEM image of the ductal end. (D) SEM image of collagen fibers interacting with ductal epithelial cells. (E) SEM image of collagen bundles wrapping around the cell in multiple directions. (F) Collagen fibrils possessing a rod-like structure with the characteristic ~67 nm banding pattern validating the presence of fibrillar collagen in proximity to epithelial cells. (G) SEM image of collagen fibrils immediately adjacent to the cell surface. (H) TEM image of collagen fibrils (col) next to the epithelial cell (e).

Examination of the ECM surrounding mammary epithelial cells revealed that the majority of the stroma is fibrillar collagen, which was seen surrounding epithelial cells (Figure 1). Figure 1A, in which picrosirius red (a selective staining agent for collagen) was used indicates the presence of collagen around the mammary ductal structure (Figure 1A,B). Observations at high magnification with SEM showed collagen fibers wrapping around the ductal structure in an organized manner surrounding the epithelial cells (Figure 1C), in a fashion similar to that seen in Figure 1A. Additionally, collagen can be seen wrapping individual cells in multiple directions (Figure 1C–E), suggesting a role for collagen as a containing and anchoring structure. Of further importance, higher magnification images with both SEM (Figure 1F,G) and TEM (Figure 1H) confirmed that the collagen fibers are composed of fibrillar collagen (Figure 1F; as indicated by their rod-like structure and presence of the ~67 nm banding pattern [32]) that are adjacent to the epithelial cells (Figure 1G,H). These data, in combination with published works indicating an important interaction between the stroma and the epithelial cell (see [4,5,33]), could suggest that these fibers apply physical restraint and mechanical signals across a thin basement membrane (<200 nm), or that the thin mesh-like basement membrane may contain some small gaps or membrane spanning cell processes and therefore may not completely isolate the epithelial cell; resulting in micro-regions of direct collagen-epithelial cell interactions [34]; or, most likely, a combination of both scenarios.

**Figure 2:**
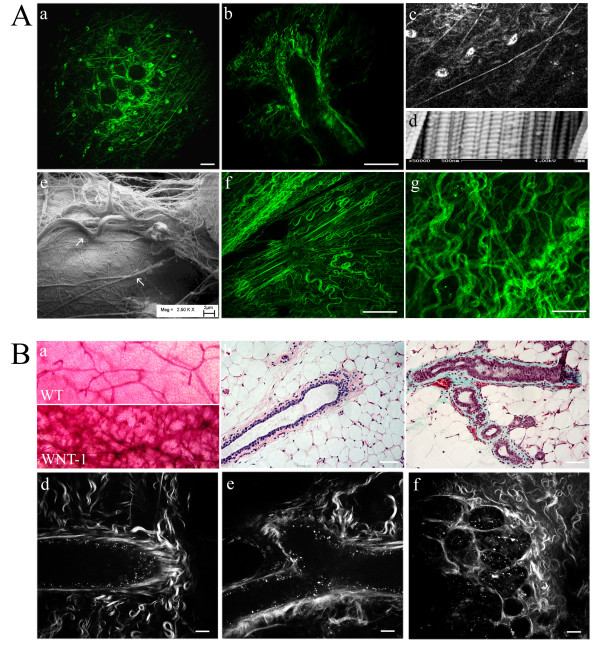
**MPLSM/SHG imaging reveals native collagen structure in living mammary gland**. A: (a) MPE/SHG image at the "top" of the mammary duct, showing both wavy and taut (straight) collagen structures as well as endogenous fluorescence from FAD in stromal cells (most likely fibroblasts and immune cells). (b) MPE/SHG image of a mammary duct demonstrating collagen wrapped around the duct as well as radiating out from the duct (as can be seen predominantly at the top of the micrograph near the end of the duct). (c) Enlarged section from (a) showing some aspects of collagen fibril structure that resemble the standard banding pattern seen in collagen fibrils from connective tissue. (d) SEM of mouse tendon mouse tendon demonstrating the ~67 nm banding pattern characteristic of collagen fibrils. (e) Correlative SEM image of collagen surrounding ductal epithelial cells showing both wavy (upper arrow) and taut (lower arrow) fibers that match the collagen structures obtained with MPE/SHG imaging. (f) MPE/SHG image of the region near the nipple in tissue demonstrating straightened collagen fibers radiating from the central ductal structure. (g) MPE/SHG image "above" the mammary duct showing both wavy and taut fiber structures. Note: all mammary tissues in A are from the B6/129 strain, which serves as the background strain for col1a1 mice. B: (a) Whole mount analysis from wild type control (top) and Wnt-1 mice (bottom), which display hyperplasia compared to wild type glands. (b,c) Histology of mammary glands from wild type control animals (b; H&E) and hyperplastic abnormal ducts from Wnt-1 mice (c; Masson's Trichrome). (d,e) MPLSM/SHG imaging of wild type control mammary duct demonstrating a ductal end (d) and branching (e). (f) MPLSM/SHG imaging of a mammary duct from a Wnt-1 mouse showing hyperplasia of the ductal structure and increased deposition of disorganized collagen. Note: All MPE/SHG images are from live intact tissues that are not fixed, sectioned, or stained; scale bar for MPE/SHG images equals 25 μm; scale bar for histology images equals 50 μm.

Although histology and electron microscopy provide valuable and detailed information regarding the composition and morphology of the epithelial-stromal interaction, these techniques are destructive to the sample and the capability for three-dimensional imaging is limited. Therefore, we have sought non-destructive imaging techniques (e.g. MPE/SHG) that allow imaging in four dimensions (x, y, z, and time). Combined MPE-SHG produced clear images of collagen (Additional file 1) as well as endogenous fluorophores, such as NAD(H) and FAD (Additional file 1, part C), in intact non-treated tissues [13,15,21]. Application of MPE/SHG to intact, non-fixed, non-stained mammary gland captured the morphology of the fibrous stroma seen in Figure 1. For example, images captured "above" the gland (Figure 2A: a and 2A: g) clearly showed the presence of wavy (crimped) collagen as well as the presence of taut (straight) fibril bundles (Figure 2A: a,e,f,g). Taut fibers showed a periodicity of ~250 nm (Figure 2A: c), consistent with previous reports for SHG resolution [14], which may represent an approximately fourfold super-periodicity of the basic ~67 nm banding pattern of collagen fibrils noted with SEM (Figure 2A: d). Additionally, collagen was observed wrapping around the epithelial duct (consistent with Figure 1A,C), as well as radiating away from the duct (Figure 2A: b). Furthermore, in addition to detecting unique features from normal mammary glands, examination of the epithelial-stromal interaction in Wnt-1 mice [35], which possessed ductal hyperplasia (Figure 2B: a–c), revealed abnormal mammary duct development with irregular collagen organization (Figure 2B: b,c). The ability to clearly discern these pathologic changes in tissues that were not fixed, sectioned, or stained (Figure 2B: d–f) validates the application of MPE/SHG imaging to detect normal mammary gland and collagen structure as well as abnormalities in mammary ducts and collagen distribution in situ (Figure 2B: f).

### Detecting dense mammary tissue

**Figure 3:**
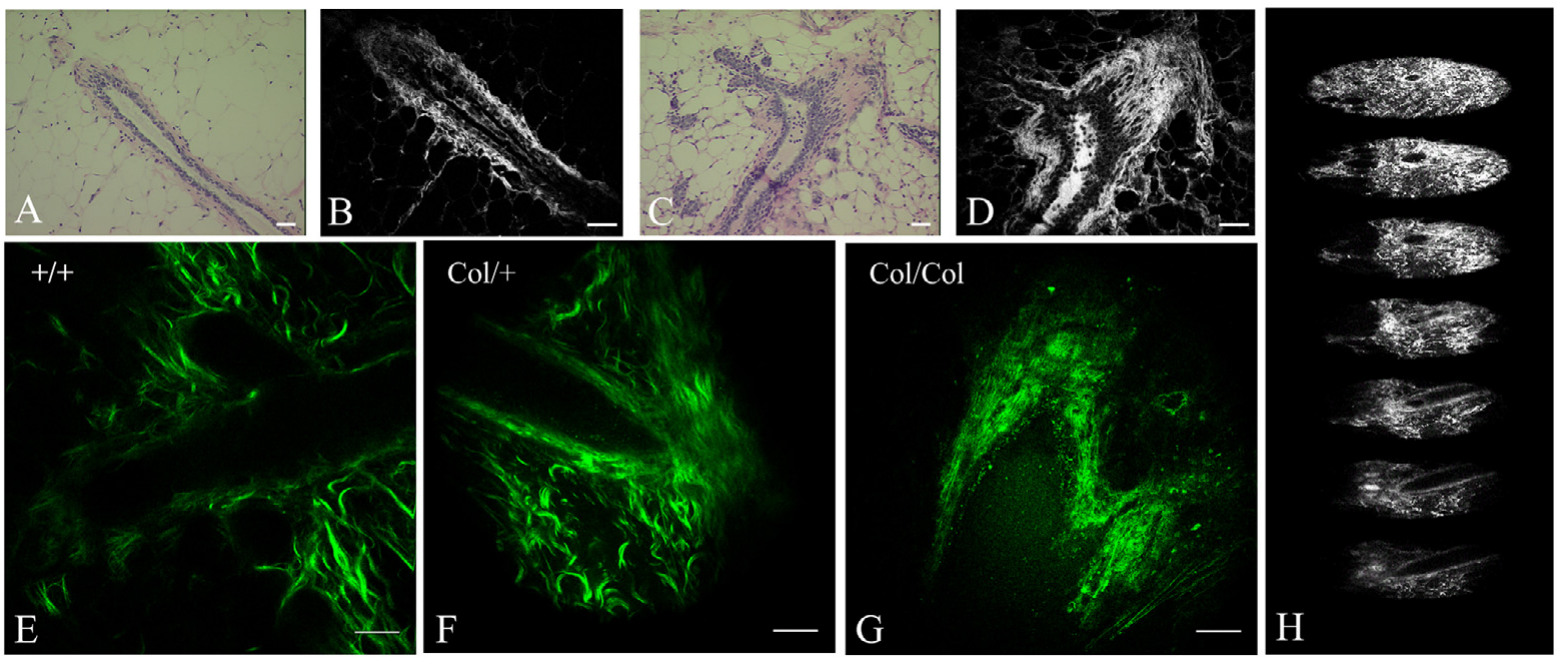
**MPLSM/SHG imaging detects increased collagen density in the mammary gland**. (A) H&E stain of wild type control mammary gland imaged with bright field microscopy. (B) MPE image of (A) showing the power of MPLSM to visualize collagen structure. (C) H&E stain of mammary gland from a col1a1/col1a1 homozygote mouse. (D) MPE image of (C) showing increased visualization of collagen density and structure and the irregular epithelial-stromal boundary, with epithelial cells invading into the locally increased collagen stroma. (E-G) MPLSM/SHG image of live mammary ducts that are not fixed, sectioned, or stained from wild type (E), heterozygous (F), and homozygous (G) col1a1 mice, showing increased collagen density in transgenic animals. (H) Z-stack of (F) spanning 340 μm (typical depth capability 350–440 μm) illustrating the structure of the glands and their relative location in the gland (see Additional file 4). Note: scale bar for MPE/SHG images equals 25 μm.

High breast tissue density (due to increased collagen; [36]) is one of the single largest risk factors for developing breast cancer [37], yet the molecular mechanisms behind this high risk are not known. One reason for this deficit has been the lack of adequate animal model systems for studying the effects of increased collagen density in vivo. As such, we sought to identify an animal model system possessing collagen-dense breast tissue. Analysis of the col1a1^tmJae ^mouse model [38] revealed such a system. These mice possess a type I collagen mutation in the α1(I) chain, making them resistant to human collagenase, resulting in fibrosis of the skin and uterus due to excessive collagen accumulation [38]. However, increases in collagen deposition in the mammary glands of these mice have not been previously described. Analysis with standard histology clearly revealed increased collagen surrounding the mammary ducts in both homozygous and heterozygous female mice regardless of parous status (Figure 3A vs 3C; Additional file 2). By utilizing histology and imaging hematoxylin and eosin (H&E) stained sections with MPLSM (SHG signal did not exist after formalin fixation; data not shown), the presence of increased collagen was confirmed (Figure 3B vs 3D), as well as an apparent hyperplasia in Col1a1 mice (Figure 3C,D; Additional file 2). Interestingly, this hyperplasia was associated with invasive-looking epithelial cells that resemble an epithelial-mesenchymal transition at the ductal end (Figure 3C,D). Therefore, in an approach opposite to that taken by Jain and co-workers [39] where a decrease in collagen in vivo was detected with SHG, we detected increased collagen in live intact mammary glands. Analysis of glands from col1a1 heterozygous and homozygous mice, and associated wild type littermates, noticeably revealed increased collagen surrounding epithelial ducts from transgenic mice (Figure 3E–H), demonstrating our ability to detect differences in collagen density. Furthermore, collagen was locally dense adjacent to (wrapped around) the epithelium, as well as increased in the space extending from the duct resulting in a 2.53- (± 0.24) and 2.79- (± 0.34) (mean ± SEM) fold increase in the area of collagen signal, as well as increased signal intensity around the duct, for heterozygous and homozygous col1a1^tmJae ^mice relative to wild type animals, respectively. Therefore, the col1a1 mouse model appears to be a promising candidate for studying the effects of increased collagen density in normal as well as transformed epithelial cells as appropriately crossed heterozygous col1a1 mice are capable of forming tumors without inhibition from the collagenase resistant matrix (P.P.P. and P.J.K unpublished observations). Moreover, we could discern changes in density and obtain images from deep (maximum Depth: 440 nm) within intact mammary tissue (Figure 3H; Additional file 4) without fixing, sectioning, or staining the tissues, suggesting that this approach could have wide application in live animal or tissue models for understanding stromal changes associated with pathologic conditions.

### Tumor-associated collagen signatures (TACS)

#### Tumor-associated collagen signature-1

**Figure 4:**
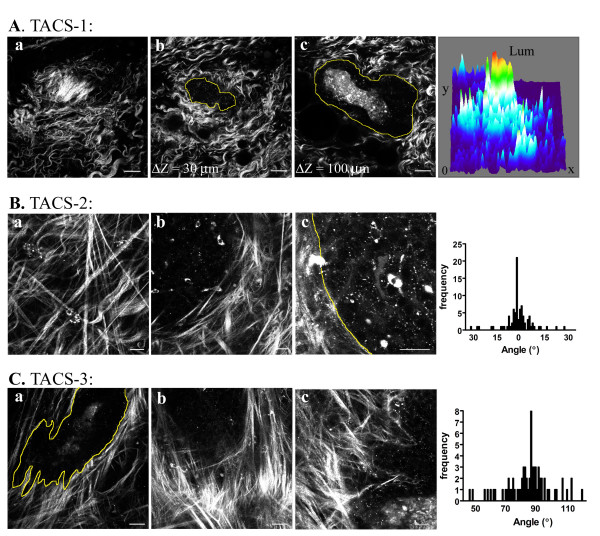
**The tumor-stromal interaction: representation of the three TACS in Wnt-1 mouse tumors**. (A-C) Micrographs illustrating the identified TACS. A: TACS-1. MPE/SHG image of TACS-1. Namely, a region of dense collagen (a, and surface map) "above" a non-palpable tumor (b,c; yellow outline) that is indicative of the presence of a small tumor (also see Additional file 5). The surface map quantifies the intensity of the fluorescent signal relative to x-y location, and clearly demonstrates an increased collagen signal, and is representative of six Wnt-1 tumors and eight PyVT tumors (not shown) B: TACS-2. MPE/SHG image of the second TACS indicated by the presence of straightened (taut) fibers characteristic of a larger Wnt-1 tumor. B: a-c, MPE/SHG images of collagen fibers in Wnt-1 mice stretched around a relatively smooth tumor boundary (outlined with a yellow line in c) as demonstrated by the fact that majority of the fibers are parallel to the tumor boundary. B: histogram, the angle of collagen fibers relative to a line tangential to the tumor boundary was measured for 86 regions in six independent tumors, and graphed as a frequency distribution resulting in a distribution of fibers around 0°. C: TACS-3. The third TACS: aligned collagen fibers at regions of cell invasion in Wnt-1 mice. C: a-c, he irregular tumor boundary associated with local invasion is outlined in yellow (in a) and connected to fibers that are primarily distributed normal to the initial tumor boundary, represented by a frequency distribution around 90° relative to the tumor boundary. C: histogram, the angle of collagen fibers relative to a line tangential to the tumor boundary was measured for 71 regions in six independent tumors, and graphed as a frequency distribution resulting in a distribution of fibers near 90°. Scale bars equal 25 μm.

Analysis of tumor bearing Wnt-1 mice, which progress through hyperplasia, mammary adenocarcinoma, and invasive (metastatic) ductal carcinoma, revealed multiple epithelial clusters, containing hemorrhagic regions, intermixed and surrounded by increased collagenous stroma (i.e. desmoplasia), consistent with previous reports [35]. Importantly, the presence of increased collagen allowed us to identify pre-palpable tumors (Figure 4A: a–c, Additional file 5; and confirmed with histology – data not shown) by the existence of what we are classifying as one of three tumor-associated collagen signatures (TACS). Namely, TACS-1: the presence of dense collagen (Figure 4A: a), indicated by increased signal intensity (see surface map in Figure 4A) at a region near the tumor (Figure 4A: a; Additional file 5) that served as a reliable hallmark for locating small tumor regions. Although increased collagen has been reported near tumors, the structure of such collagen has previously been largely unknown, and the additional focal localization shown in Figure 4A indicates increased local areas of high density within the globally increased collagen concentration surrounding tumors. However, it is unclear whether: (1) a pre-tumor dense region is present that serves to stimulate tumor formation, (2) the dense region of collagen is pulled into a grouped cluster through increased contraction of an epithelial tumor mass or motile cells at the tumor boundary, or (3) fibroblast activation results in increased local collagen deposition. Further analysis into this fundamental characteristic should provide useful information on tumor initiation and progression.

#### Tumor-associated collagen signatures-2 and -3

As the size of the tumor increased, we identified a second collagen signature, TACS-2: "taut" collagen fibers stretched around the tumor (Figure 4B). This collagen morphology likely arose from stretching of the stroma due to tumor growth, which may act to constrain portions of the tumor (i.e. compressive restraint) as well as provide a stretch induced tensile stress in expanded fibrils (and larger resistance to cell contraction in the stroma) that stimulates and activates fibroblasts. Evidence for tumor restraint in Wnt-t mice can be seen in Figure 4B, a–c, where collagen fibers are stretched around a relatively smooth tumor boundary as indicated by the fact that the fiber angle is primarily distributed tangentially (0° relative to the tumor boundary) along the tumor boundary (see histogram in Figure 4B).

In regions of tumor masses that are undergoing growth and invasion (Figure 4C: a–c), a third tumor-associated collagen signature, TACS-3, was identified: collagen fibers aligned normal to tumor boundary regions that display an irregular shape – indicative of local invasion through collective epithelial cell migration [22,40]. This invasive tumor morphology was seen in regions of tumors where collagen fibers are primarily aligned in the direction of cell invasion (see histogram in Figure 4C, in which the angle of the collagen fibers relative to the tumor boundary distributes around 90°). Furthermore, at regions where TACS-3 is noted, we observed groups of cells advancing from the tumor boundary in a collective manner that appear to be undergoing collective invasion [22,40,41], as well as individual invading cells that may relate to single cell migration similar to what has been observed along collagen fibers in an in vivo xenograft model [9].

**Figure 5:**
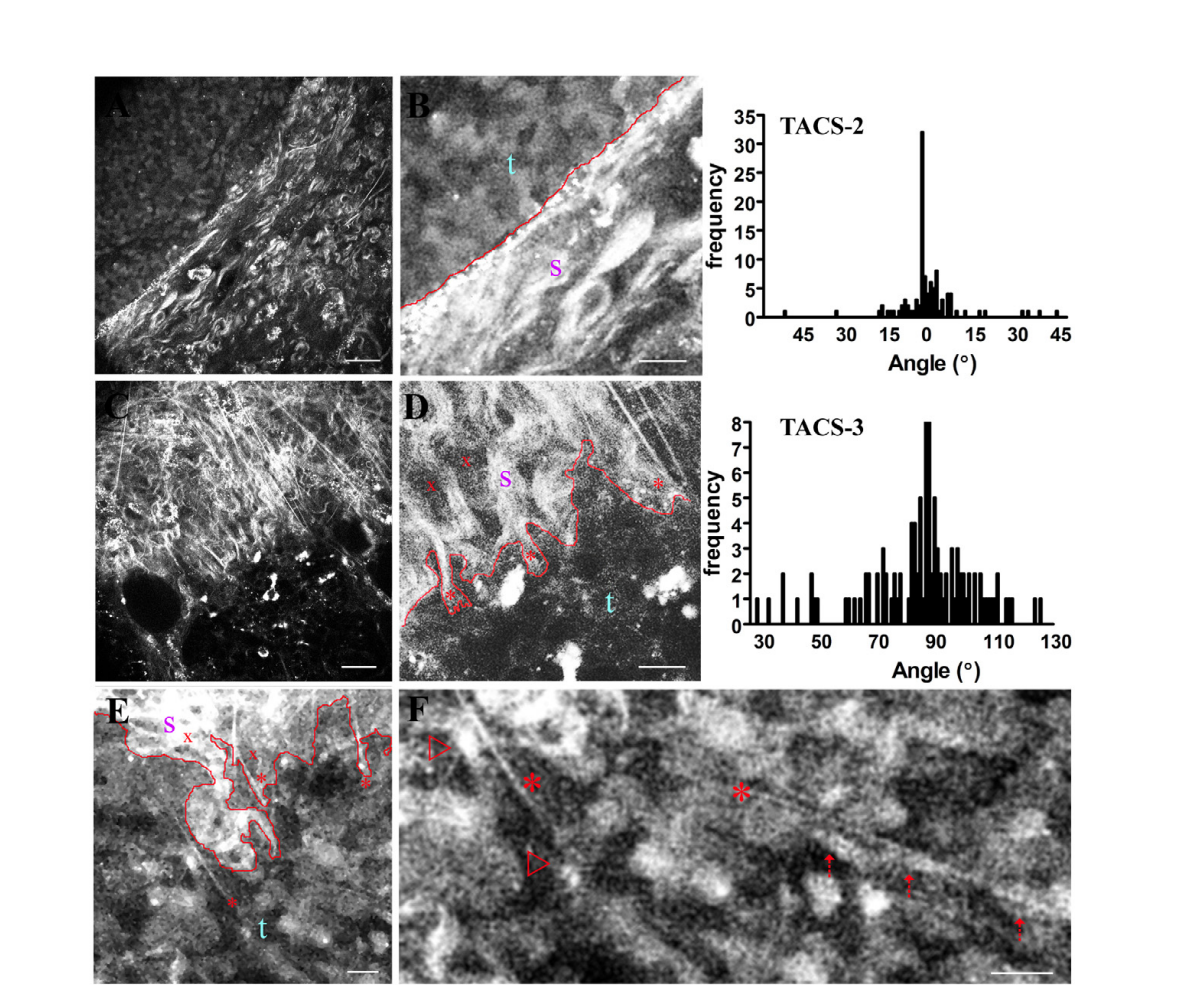
**Study of TACS-2 and -3 in the more aggressive PyVT mouse tumor model**. (A) TACS-2, with an enlarged cutout region (B) shown at higher brightness and contrast levels and a rough demarcation of the tumor-stromal boundary (red line; s = stroma; t = tumor) to further indicate the wrapping of the collagen parallel to the tumor boundary (distribution near 0°, see top right TACS-2 histogram of 106 regions from eight independent tumors). (C) TACS-3, with an enlarged cutout region (D) shown at higher brightness and contrast levels and a rough demarcation of the tumor-stromal boundary (red line; s = stroma; t = tumor). Note that although some cells have moved past this boundary (examples = x) into the fibers, the boundary serves as a general representation of irregular invasive region into radially aligned collagen fibers (frequency distribution around 90°, see middle right TACS-3 histogram of 109 regions from eight independent tumors). Furthermore, analysis of TACS-3 regions (E) at higher magnification (F) show endogenous cells associated with fibers at the tumor-stromal boundary and within the tumor. * Indicates examples of fibers interdigitated with the invasive tumor boundary and in contact with the invading tumor cells (red arrows). Scale bar for MPE/SHG images A, C, E, and F equals 25 μm, and 10 μm for B and D.

**Figure 6:**
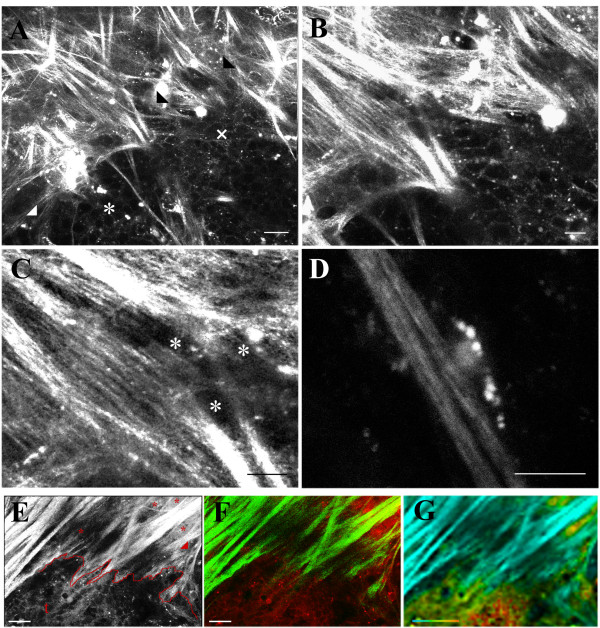
**TACS-3 – radially aligned collagen fibers associated with invasion**. Combined MPE/SHG imaging of live intact PyVT mammary tumors indicates that (A) non-invading regions (*) possess taut collagen (TACS-2; white arrowhead) wrapped around the tumor, while regions of invasion (x) are linked to aligned collagen, with cells invading along radially aligned collagen fibers (TACS-3; black arrowheads). Examination of invasion at the tumor-stromal interface at higher magnification (B and C) clearly reveals the collagen alignment at specific regions of invasion (B) with tumor cells (*) in between, and in association with, aligned collagen fibers (C). (D) Example of an individual tumor cell attached to a collagen fiber leading away from the primary tumor. Additionally, tumor cells that have invaded across the tumor-stromal boundary can be visualized by separating the MPE and SHG signals or imaging with FLIM. (E) Combined MPE/SHG image of a TACS-3 region facilitating local invasion. The tumor-stromal boundary is not well preserved at this stage, but is roughly outlined in red. Examples of regions of cells that have invaded past the boundary are marked with an asterisk (t = the primary tumor). The red arrowhead indicates cells near the tumor boundary that are migrating along aligned collagen fibers away from the primary tumor. (F) MPE and SHG signal separation of the image shown in E. MPE signal is represented in red pseudo-color while SHG is shown in green pseudo-color. Note the interdigitation of aligned collagen fibers (green) into the tumor, with individual, or lines of, cells (* in E) migrating away from the tumor on collagen fibers. (G) FLIM micrograph of invading cells at the TACS-3 region shown in E and F. Collagen (blue; no fluorescence lifetime) can be distinguished from cells (green to yellow), confirming the presence of invading cells at the tumor-stromal boundary and cells that have migrated past the boundary in association with collagen. The color bar in G ranges represents the weighted mean ranging from 100 ps (blue) to 1 ns (red). Scale bars equal 25 μm in A, E, F, and G; 10 μm in B, C, and D.

To further investigate the behavior of the third collagen signature in a more invasive and metastatic cancer model, we utilized the well established and characterized PyVT mouse model. This model bears resemblance to many aspects of human cancer, is reliably invasive and metastatic following defined, progressive, and reliable histological grades from hyperplasia to adenoma, and then to early and late carcinoma, and therefore provides a good model for studying human disease [42]. Tumor cell behavior and collagen morphology in this model were similar to observations from the Wnt-1 mouse tumors. For instance, tracking the tumor-stromal boundary around individual tumors (Figure 5A–D) first revealed regions of TACS-2 morphology, in which restraining collagen wrapped around a relatively smooth boundary (Figure 5A,B, and histogram: TACS-2). In regions of TACS-2, cells had a mostly random alignment with a subset of cells aligned in the same direction as the collagen (Figure 5A,B). The second region revealed a TACS-3 boundary where collagen has been primarily aligned perpendicular to the tumor (Figure 5C,D, and histogram: TACS-3). A reasonable hypothesis for this behavior is that collagen fiber realignment occurs through morphogenesis and contractile events from cells at the tumor boundary, to organize the matrix and to prepare for local invasion (see Figure 6 for TACS-2 to -3 transition). Evidence for this hypothesis is seen by the collagen structure associated with regions of invading cells in the Wnt-1 tumors (Figure 4C), PyVT tumors (Figure 5A–D; Figure 6), and MPLSM analysis of invasive regions in tumor histology sections (Additional file 3). In all these cases local tumor invasion occurred where collagen fibers are aligned radially from the tumor in the direction of tumor cell invasion (see TACS-3 histogram in Figure 5, and the change in stromal alignment correlated with the change to invasive cell morphology in Figure 6). Additionally, some invading cells were in direct contact with fibers (Figure 5E,F; Figure 6C,D). In certain cases, some matrix disorganization was also observable (see Figure 4C: c and Figure 5E) possibly indicating proteolytic cleavage of collagen [40,43].

**Figure 7:**
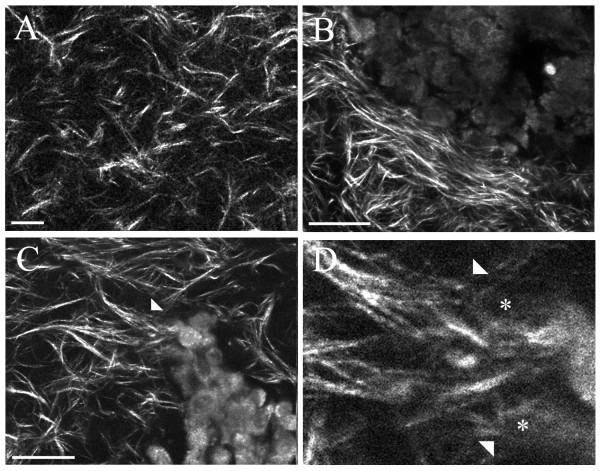
**Collagen matrix reorganization by tumor cells facilitates local invasion of tumor cells**. Combined MPE/SHG imaging of tumor explants cultured within 3D collagen gels for eight hours demonstrates that tumor cells reorganize a previously random matrix to facilitate invasion. (A) Region of 3D collagen gel remote from the tumor demonstrating the random orientation of collagen present within 3D collagen gels in a region that has not been reorganized by a specific outside force. This random organization is specifically altered by cells from tumor explants as the cells contract and reorganize the collagen matrix. Similar to data in live intact tissues, non-invading regions show collagen pulled in near the explant but wrapped around the tumor boundary (B), while at regions of tumor cell invasion into the collagen gel, collagen has been radially aligned by the tumor cells (white arrow heads C) with cells (*) in direct contact with the collagen matrix (white arrowheads; D). Therefore, at regions of tumor cell invasion, collagen has been reorganized to a radial alignment from a random orientation, indicating a structural realignment of collagen fibers facilitates local invasion. Scale bar equals 25 μm.

To further test the hypothesis that tumor cells realign the collagenous matrix to facilitate local invasion, we determined whether tumor cells could reorganize a random collagen matrix. Analysis of tumor explants within type-I collagen gels revealed radial collagen alignment at regions of tumor cell invasion into previously randomly aligned collagen gels (Figure 7), consistent with previous reports analyzing fixed tumor explants in 3D reconstituted matrices [22], and supporting information about invasion within intact live tissues (Figures 4, 5, 6). Following polymerization, circular collagen gels (disks) displayed a random orientation of collagen fibers (Figure 7A) without an outside force to initiate structural reorganization. Tumor explants cultured within such random collagen gels contracted and reorganized these collagen gels, resulting in various local outcomes with collagen wrapped around the explant at non-invading regions (Figure 7B). In contrast, at regions of invasion into the gels, collagen was reorganized to a radial alignment (Figure 7C) with direct contact between collagen fibers and invading cells (Figure 7C,D). This demonstrates that tumor cells can de novo reorganize a random collagen matrix to facilitate invasion, supporting the hypothesis that matrix reorganization at the tumor interface facilitates local invasion in vivo. Hence, our results suggest that in order to facilitate local invasion, cells at the tumor boundary contract and align collagen fibers, perhaps with the assistance of proteolytic cleavage to facilitate matrix reorganization, and then invade along aligned collagen structure to expand the tumor and later metastasize.

## Discussion

Intrinsic fluorescence detection with multiphoton excitation in combination with SHG facilitates three-dimensional, high resolution, imaging in unfixed, unsectioned, unstained mammary tissues. This imaging provides information commonly obtained with classical histology and EM without the need for complex and destructive sample preparation, and with additional structural information in four dimensions. With MPE/SHG, mammary gland tissue could be clearly imaged at depths of 440 nm, and changes in collagen density could be reliably detected. Furthermore, three-dimensional imaging of tumors in situ revealed three now defined TACS, which provide standard hallmarks to locate and characterize tumors: TACS-1, the presence of dense collagen, indicated by increased signal intensity at a region around the tumor as a standard hallmark for locating small tumor regions; TACS-2, the presence of taut (straightened) collagen fibers stretched around the tumor, indicating growth leading to increased tumor volume; and TACS-3, the identification of radially aligned collagen fibers facilitating invasion, which may be indicative of the invasive and metastatic growth potential of a tumor. Together these signatures may serve a mechanism to help identify and characterize breast tumors in experimental animal models as well as human cancers and fresh tumor biopsies.

The breast epithelial cell-ECM interaction is responsible for influencing cell polarity, proliferation, differentiation, adhesion, and migration [44,45] and type I collagen is an important regulator of mammary ductal formation during development [4]. Analysis of normal mammary glands reveals collagen fibers wrapping around, as well as radiating away from, the duct (Figure 2A: b). This organization is remarkably consistent with the observation that in fixed whole mounts of developing mammary gland, analyzed with multiphoton microscopy, collagen fibers are "pulled in" perpendicular to the terminal end bud [46], similar to what we observe for radially aligned collagen fibers near tumors (TACS-3). Combined, these morphologies provide insight into the structure-function relationship in the mammary gland and imply that collagen may provide directional cues during development that also influence changes in the normal mammary gland. For instance, the crimped (wavy) collagen structure (i.e. Figure 2A: a,b,e,g) is consistent with numerous reports of crimped collagen fibers in connective tissue that allow normal tissue deformation with a strain-stiffening behavior [47,48]. This behavior may hold true for the mammary gland as well, allowing for tissue deformation and normal ductal growth and involution without over constraining the system, yet providing adequate levels of tensile resistance to contracting cells and resisting large deformations that can damage the tissue. The less numerous taut fibers may serve a different purpose. They may act as locally constraining structures at the single cell level and may act to interconnect various ducts in the tissue together and to the nipple structure (Figure 2A: f), which may transmit mechanical signals to the ducts during activities such as nursing to elicit mechanotransductive signaling related to lactation. Furthermore, such mechanical signals acting directly on epithelial cells or transmitting stress across the basement membrane would be amplified by increased breast tissue density. Hence, increased breast tissue density in vivo may promote carcinoma formation by increased mechanical signaling events in dense tissue, consistent with in vitro work showing that increased matrix density alters breast epithelial cell signaling [49].

The importance of matrix composition and morphology around the mammary epithelium is illustrated by studies showing that misregulated stromal-epithelial interactions can promote tumorigenesis [6-8] and the fact that breast carcinomas often exhibit desmoplasia (excessive collagen surrounding an invasive tumor [50]). Moreover, cancer cells can locally invade across basement membrane and collagenous stroma to spread into neighboring ECM environments, where they can migrate further to enter lymphatic and blood vessels, resulting in metastatic growth in distant tissues [40,51]. Therefore, understanding the mechanisms of invasion in vivo is of great importance. Yet, to our knowledge, no study has visualized local invasion in endogenous tumors in vivo in relation to stromal organization. Consequently, it is noteworthy that we observe alignment of collagen fibers, and association of individual cells with those fibers at regions of local invasion in live tissue (TACS-3), which is similar to observations of individual cell migration along collagen fibers in a xenograft model [9], and confirms and expands upon in vitro studies in 3D matrices that have identified collagen reorganization (alignment) at the front of invading cells [22,40,41]. Moreover, the concept of alignment-facilitated invasion appears to be of significance in collective cell migration (e.g. tubulogenesis in the mammary gland; [41]) as collagen alignment is noted at the terminal end bud during invasion of the mammary ductal tree [46]. Thus, collagen alignment may facilitate motility and migration during normal development, while tumor invasion may resemble misregulated developmental processes.

## Conclusion

The data presented indicate that tumor cells often localize near dense collagen or promote a desmoplastic response and contract and localize collagen, followed by tumor growth and expansion (stretching) of the collagen matrix leading to matrix reorganization (possibly assisted by proteolytic cleavage [40,43] to release collagen fibers) to help facilitate local invasion. This matrix reorganization would require enhanced contractility and motility of the tumor cells, which may explain the increased presence of Rho and ROCK, in invasive cancers ([52], and references therein). Although a number of mechanisms, such as growth factor and integrin signaling and protease secretion and activity, are associated with invasion and metastasis, it seems likely that GTPase-regulated motility events are also involved these processes. Hence, the mechanisms behind local invasion may include matrix reorganization through GTPase-mediated tumor cell contractility (P.P.P. and P.J.K unpublished observations), leading to an aligned matrix that facilitates local invasion.

## Competing interests

The authors declare that they have no competing interest. However, portions of the technologies presented in the manuscript are patent pending. The authors have no interest, arrangement, or affiliation that could be perceived as a conflict of interest in the context of this manuscript.

## Authors' contributions

PPP conducted all MPLSM, SHG, SEM, and histology experiments, managed mouse colonies, performed 3D cell culture experiments, analyzed the imaging data, and prepared the manuscript and figures. DRI assisted with mice and performed 3D culture experiments. JMC performed TEM imaging of mouse glands. KWE and JGW assisted with specific technical aspects of nonlinear imaging and data analysis as well as project coordination. PJK participated in the design and coordination of the project and assisted with data analysis. PPP, KWE, JGW, and PJK cooperatively designed the project and discussed data interpretation and analysis. All authors participated in critical editing of the manuscript and read and approved the final manuscript.

## Pre-publication history

The pre-publication history for this paper can be accessed here:



## Supplementary Material

Additional File 1 MPM/SHG imaging of collagen and endogenous fluorescence in connective tissue and reconstituted 3D matrix.  **(A) **SHG image of type I collagen in mouse Achilles tendon. **(B) **MPM/SHG image of cellular endogenous fluorescence and collagen in skin. **(C)**Collagen and NADP(H) detection with combined MPM/SHG imaging of mouse pectoral muscle. **(D)** SHG imaging of type I collagen in a reconstituted three-dimensional gel.. Click here for file

Additional File 2 Col1a1tmJae mice possess collagen dense mammary tissue.  Histology of homozygous col1a1 mice **(A)** showing increased collagen surrounding the mammary duct as detected with H&E trichrome, and picrosirius red (Picro) staining. **(B)** Increased collagen is also present in heterozygous col1a1 mice as detected with H&E; and picrosirius red staining (PS).
Click here for file

Additional File 4 Movie file showing MPE/SHG imaging of collagen surrounding the mammary duct at >440 μm into the live tissue. Click here for file

Additional File 5 Movie showing MPE/SHG imaging of TACS-1. Click here for file

Additional File 3 MPLSM examination of TACS-2 and TACS-3 in H&E sections of PyVT tumors.  Left Column: Non-invading region of the tumor showing TACS-2 (see middle left panel arrows) that can be confirmed with MPLSM (bottom left).  Right Column: Invading region of the tumor showing TACS-3 (see middle right panel arrows for examples of invading cells) that can be detected and confirmed with MPLSM (botton right).  Boxes in top row indicate region examined in the middle panels.
Click here for file
