# Supplementary material for: Collagen reorganization at the tumor-stromal interface facilitates local invasion
Source: BMC Med. 2006 Dec 26;4:38. doi: 10.1186/1741-7015-4-38 (PMC1781458; doi:10.1186/1741-7015-4-38)
Supplement: Additional File 1 — MPM/SHG imaging of collagen and endogenous fluorescence in connective tissue and reconstituted 3D matrix. (A) SHG image of type I collagen in mouse Achilles tendon. (B) MPM/SHG image of cellular endogenous fluorescence and collagen in skin. (C)Collagen and NADP(H) detection with combined MPM/SHG imaging of mouse pectoral muscle. (D) SHG imaging of type I collagen in a reconstituted three-dimensional gel.. [file 1741-7015-4-38-S1.pdf]

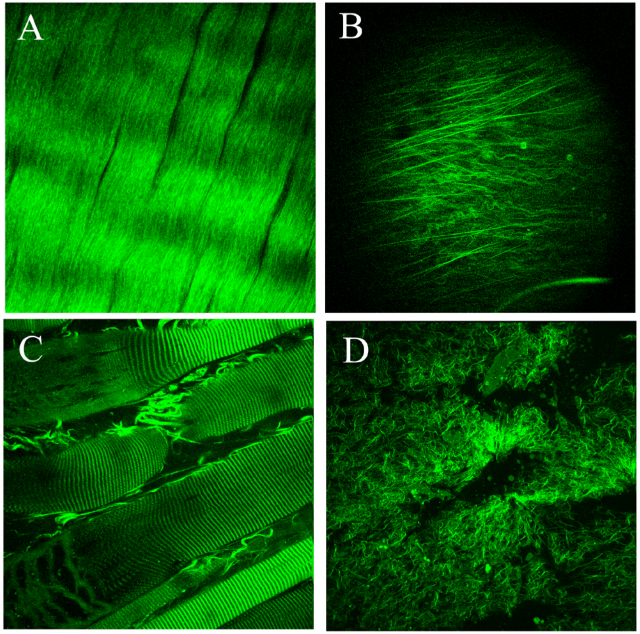

Supplementary Figure 1: MPM/SHG imaging of collagen and endogenous fluorescence in connective tissue and reconstituted 3D matrix. (A) SHG image of type I collagen in mouse Achilles tendon. (B) MPM/SHG image of cellular endogenous fluorescence and collagen in skin. (C) Collagen and NADP(H) detection with combined MPM/SHG imaging of mouse pectoral muscle. (D) SHG imaging of type I collagen in a reconstituted three-dimensional gel.
