# Supplementary material for: Collagen reorganization at the tumor-stromal interface facilitates local invasion
Source: BMC Med. 2006 Dec 26;4:38. doi: 10.1186/1741-7015-4-38 (PMC1781458; doi:10.1186/1741-7015-4-38)
Supplement: Additional File 2 — Col1a1tmJae mice possess collagen dense mammary tissue. Histology of homozygous col1a1 mice (A) showing increased collagen surrounding the mammary duct as detected with H&E trichrome, and picrosirius red (Picro) staining. (B) Increased collagen is also present in heterozygous col1a1 mice as detected with H&E; and picrosirius red staining (PS). [file 1741-7015-4-38-S2.pdf]

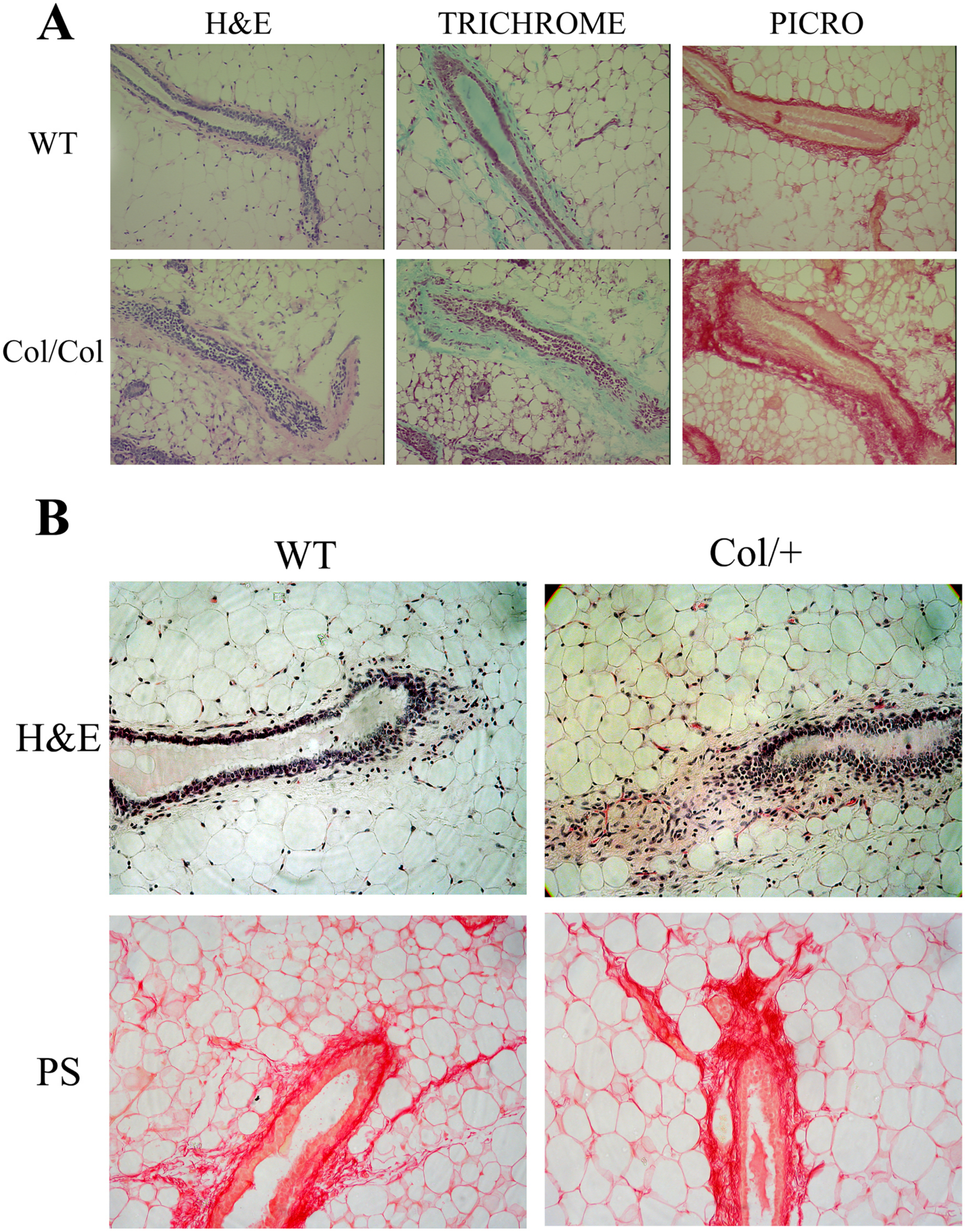

Supplementary Figure 2: Col1a1tmJae mice possess collagen dense mammary tissue. Histology of homozygous col1a1 mice (A) showing increased collagen surrounding the mammary duct as detected with H&E, trichrome, and picrosirius red (Picro) staining. (B) Increased collagen is also present in heterozygous col1a1 mice as detected with H&E and picrosirius red staining (PS).
