# Supplementary material for: Collagen reorganization at the tumor-stromal interface facilitates local invasion
Source: BMC Med. 2006 Dec 26;4:38. doi: 10.1186/1741-7015-4-38 (PMC1781458; doi:10.1186/1741-7015-4-38)
Supplement: Additional File 3 — MPLSM examination of TACS-2 and TACS-3 in H&E sections of PyVT tumors. Left Column: Non-invading region of the tumor showing TACS-2 (see middle left panel arrows) that can be confirmed with MPLSM (bottom left). Right Column: Invading region of the tumor showing TACS-3 (see middle right panel arrows for examples of invading cells) that can be detected and confirmed with MPLSM (botton right). Boxes in top row indicate region examined in the middle panels. [file 1741-7015-4-38-S3.pdf]

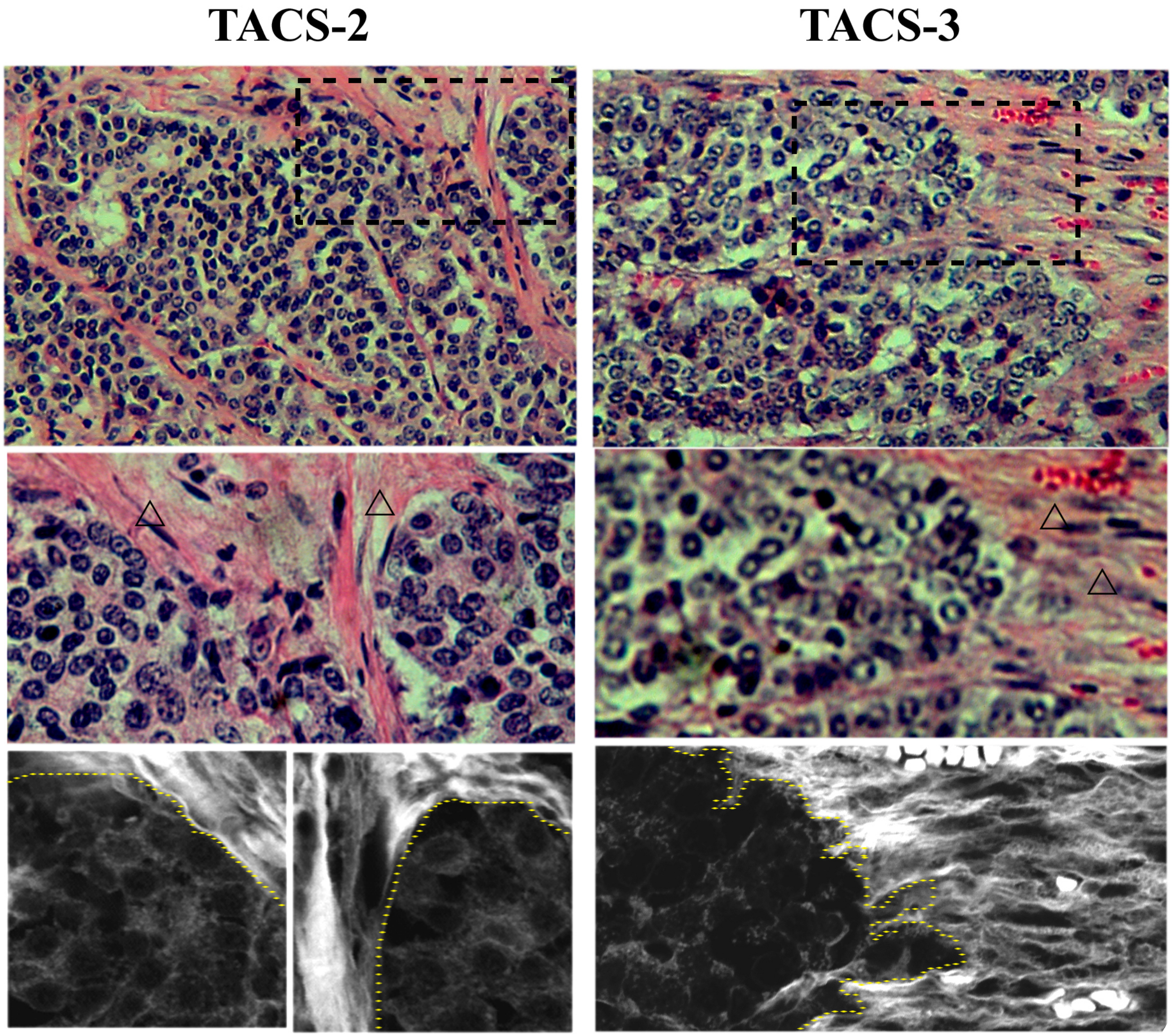

Supplementary Figure 3: MPLSM examination of TACS-2 and TACS-3 in H&E sections of PyVT tumors. *Left Column*: Non-invading region of the tumor showing TACS-2 (see middle left panel arrows) that can be confirmed with MPLSM (bottom left). *Right Column:* Invading region of the tumor showing TACS-3 (see middle right panel arrows for examples of invading cells) that can be detected and confirmed with MPLSM (botton right). Boxes in top row indicate region examined in the middle panels.
